# Exosomes in esophageal cancer: a promising frontier for liquid biopsy in diagnosis and therapeutic monitoring

**DOI:** 10.3389/fphar.2024.1459938

**Published:** 2024-12-17

**Authors:** Ren Zihan, Cao Jingsi, Ding Lingwen, Liu Xin, Zhang Yan

**Affiliations:** ^1^ Department of Thoracic Surgery, Organ Transplantation Center, The First Hospital of Jilin University, Changchun, Jilin, China; ^2^ Department of Respiratory and Critical Care Medicine, The Second Hospital of Hebei Medical University, Shijiazhuang, Hebei, China; ^3^ Department of Vaccination Clinic, Xiangyang Center for Disease Control and Prevention, Xiangyang, Hubei, China

**Keywords:** exosomes, liquid biopsy, esophageal cancer, early diagnosis, adjuvant therapy

## Abstract

Esophageal cancer is a common and lethal digestive system malignancy, and both treatment efficacy and patient survival rates face significant challenges. In recent years, exosomes have emerged as crucial mediators of intercellular communication, demonstrating tremendous clinical potential, particularly in the diagnosis, treatment, and prognostic evaluation of esophageal cancer. These exosomes not only serve as biomarkers for early diagnosis and prognosis but also modulate tumor growth, metastasis, and drug resistance by delivering bioactive molecules. Importantly, exosomes can act as carriers for esophageal cancer-related therapeutic agents, optimizing gene therapy strategies to enhance efficacy while reducing toxicity and side effects. Despite facing challenges in clinical applications such as purification, enrichment, and standardization of analytical methods, exosomes maintain broad prospects for application in esophageal cancer treatment, with the potential to significantly improve patient outcomes and quality of life. This review focuses on the innovative role of exosomes in the early diagnosis of esophageal cancer, exploring their application value and safety in disease monitoring and assessment of treatment response. Furthermore, this study outlines the challenges and limitations of transitioning exosome research from basic studies to clinical applications, as well as potential solutions and future research directions to address these obstacles.

## 1 Introduction

According to statistics from the International Agency for Research on Cancer of the World Health Organization (Sung et al., 2021), there were approximately 604,100 new cases of esophageal cancer (EC) worldwide in 2020, accounting for 3.1% of all cancer cases and ranking 7th for all cancers. The estimated number of deaths from EC in 2020 was approximately 544,076, accounting for 5.5% of all cancer-related deaths and ranking 6th sixth among malignant tumors. More concerning is that over half of the global EC cases occur in China, accounting for 53.7% of new cases and 55.3% of deaths (Zhou et al., 2019). Although in the latest global cancer statistics report (Bray et al., 2024), the incidence rate of EC had dropped to 11th place, it remains the 7th highest mortality cause of mortality worldwide. Globally, esophageal squamous cell carcinoma (ESCC) is the most common subtype, accounting for approximately 85% of all cases. Esophageal adenocarcinoma (EAC) accounts for approximately 14% (Morgan et al., 2022). There are significant differences in the incidence rates of EC among different populations and regions. Generally, the incidence rate in men is higher than that in women (Chen et al., 2023a). In terms of geographical distribution, the incidence rate of EAC is the highest in Northern Europe and North America, whereas that of ESCC is higher in South Africa, Central and South Asia, and East Asia (Dawsey and Duits, 2023; GBD 2017 Oesophageal Cancer Collaborators, 2020). The main pathological type of EC in China is ESCC, with a 5-year survival rate of approximately 30% and a poor prognosis (Li et al., 2017).

Precancerous EC lesions exhibit obvious pathological features, progressing slowly from early precancerous lesions to invasive cancers (Gao et al., 2023). A follow-up study of 1,183 patients with precancerous lesions of EC found that only 88 patients (7.44%) progressed to advanced tumors (Li et al., 2023a). This characteristic provides an opportunity for the early diagnosis and treatment of EC. Unfortunately, the onset of EC is often insidious. Patients with precancerous lesions in the early stages (stage 0, I, and II) may not exhibit obvious symptoms. However, by the time a diagnosis is established, the disease often progresses to an advanced stage, resulting in missed opportunities for optimal surgical treatment. Therefore, it is crucial to develop a highly sensitive and specific diagnostic tool for the screening and early diagnosis of EC in high-risk populations.

Liquid biopsy is an innovate approach for the diagnosis of diseases including cancer by examining the biomarkers in blood or other body fluids. Liquid biopsy has garnered significant attention in global cancer research (Markou et al., 2022). The technology was recognized as one of the top ten breakthrough technologies by the Massachusetts Institute of Technology Review magazine in 2015 because of its speed, low invasiveness, and good patient compliance. The significance of liquid biopsy in the early diagnosis, drug monitoring, and prognostic assessment of solid tumors has become increasingly evident.

Traditional tumor liquid biopsy is primarily divided into two categories: circulating tumor cells and circulating tumor nucleic acids. However, both approaches have drawbacks that include low detection rate, low specificity, and significant individual variability (Zhu et al., 2023a). The importance of extracellular vesicle, which encompass different subtypes such as exosomes (EVs), was highlighted in 2013 with the awarding of the Nobel Prize in Physiology or Medicine, thereby propelling research in this field. Among extracellular vesicle, EVs are a specific subtype, typically ranging from 30 to 150 nm in size, with a protective phospholipid bilayer membrane rich in membrane proteins that enhances the stability of the bioactive molecules they carry, such as DNA, RNA, proteins, and metabolites (Kimiz-Gebologlu and Oncel, 2022) (Arya et al., 2024). Due to these characteristics, EVs are particularly well-suited for biomarker research and hold significant promise in liquid biopsy assays.

Physiologically, exosomes are nanoscale vesicles secreted by various cells. Their payloads of various biological macromolecules that include proteins, nucleic acids, and lipids confer critical roles of EVs in the development and progression of tumors, particularly in the formation of the tumor microenvironment, facilitating communication among tumor cells and interactions between tumors and host cells (Preethi et al., 2022). Notably, EVs can circulate throughout the body via blood and other bodily fluids, and thus are a potential source of non-invasive tumor markers (Li et al., 2021a).

**FIGURE 1:**
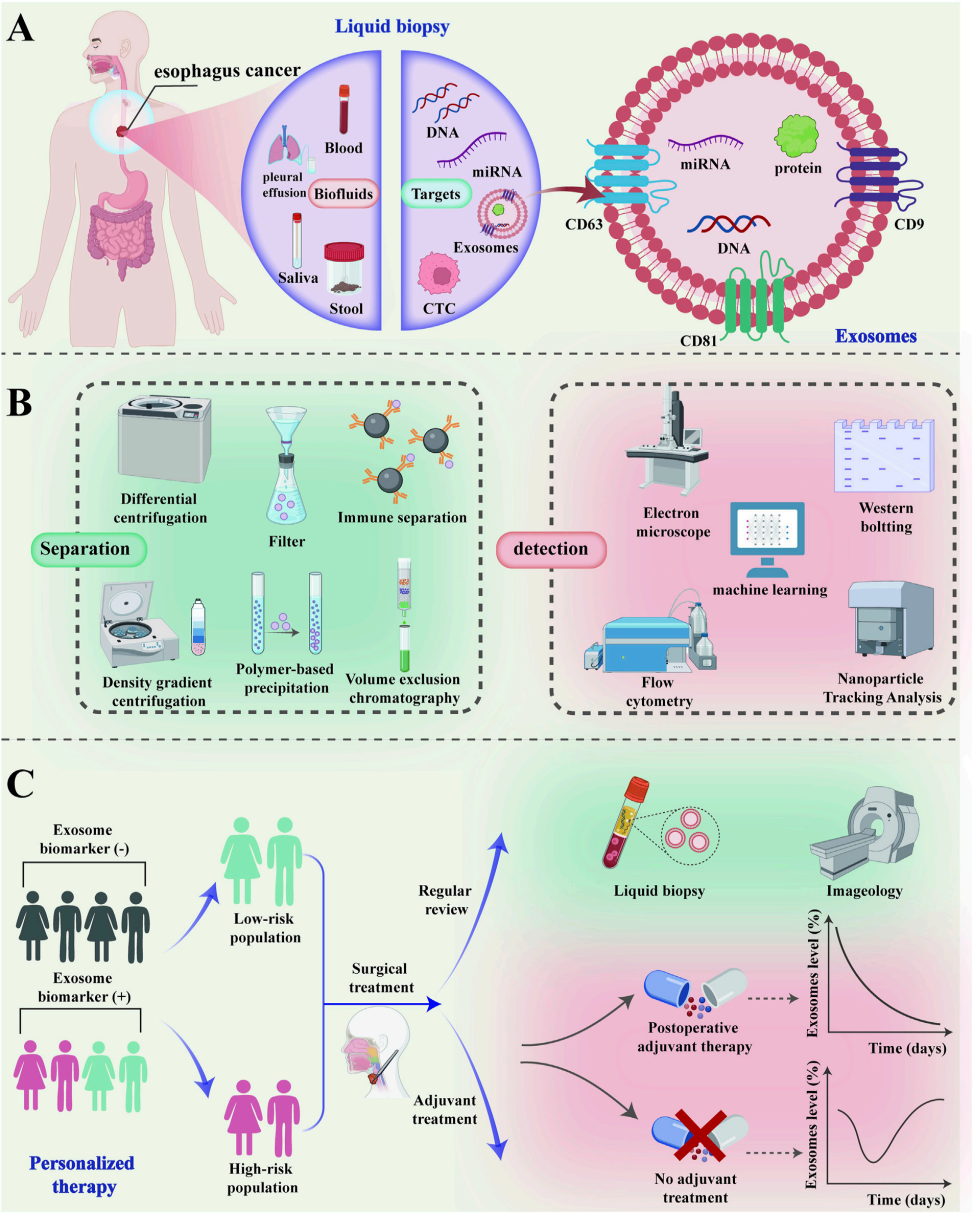
The clinical application of exosomes in the diagnosis and treatment of esophageal cancer. **(A)** As a “new star” of liquid biopsy, exosomes can be used in the diagnosis and treatment of esophageal cancer through a variety of body fluids; **(B)** The common isolation and detection techniques of exosomes; **(C)** Exosomes can be used for screening and risk stratification of high-risk groups of esophageal cancer for personalized precision treatment. The low-risk population of esophageal cancer can be monitored for recurrence by imaging examination and liquid biopsy after surgical treatment. Exosomes can be quantitatively analyzed to monitor the efficacy and prognosis of adjuvant therapy in high-risk patients with esophageal cancer after surgical treatment. CTC, Circulating Tumor Cell; miRNA, microRNA; CT, Computed Tomography.

This comprehensive review addresses the diagnosis and treatment of EC using exosomes based on liquid biopsy (Figure 1). The review delves into the relationship between liquid biopsy and exosomes, details the primary isolation and detection techniques for exosomes, and discusses the latest research on exosomes in the early diagnosis and treatment of EC in recent years. Future clinical applications of exosomes are discussed and research directions, limitations, and technical challenges are analyzed.

## 2 Relationship between exosomes and liquid biopsy

**FIGURE 2:**
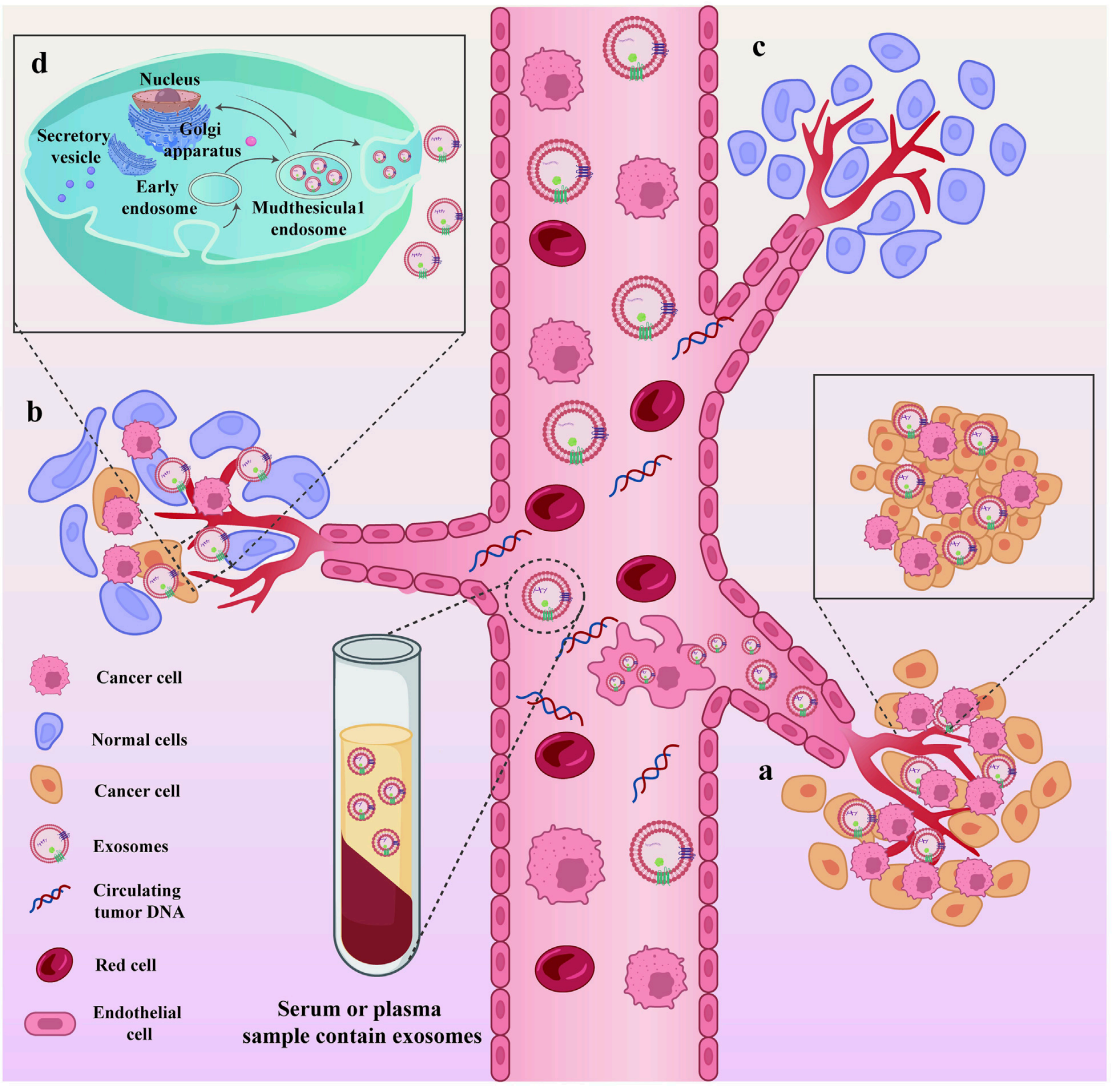
Exosomes, as cell-cell messaging systems, are involved in tumor cell growth and metastasis. Exosomes, as cell-cell messaging systems, are involved in tumor cell growth and metastasis. **(A)** During the progression of the disease, the primary tumor site continuously secretes exosomes and sheds necrotic tumor cells into the peripheral circulation; **(B)** Exosomes act as mediators of information transfer, facilitating the metastasis of tumors from the primary site to other organs; **(C)** Sites within tumor patients where metastasis has not occurred; **(D)** The process of exosome secretion within cells.

The liquid biopsy technology primarily involves the detection of free circulating tumor cells, circulating tumor DNA, and exosomes, which are used for early screening, personalized treatment, efficacy monitoring, and recurrence monitoring in patients with cancer (Amato et al., 2023). Compared to traditional tissue biopsy, the unique advantages of liquid biopsy include non-invasive, real-time dynamic monitoring, means of overcoming tumor heterogeneity, and ability of comprehensive detection information (Wu et al., 2023; McLaren and Aitman, 2023; Takahashi et al., 2023). Exosomes (Zhao et al., 2021a) are extracellular vesicles released by cells into the extracellular space. They vary between 30 and 150 nm in size, have a double-layer membrane structure, are saucer shaped, and contain a variety of components including nucleic acids, proteins, and lipids. Serving as crucial signaling molecules, EVs contribute to a novel cell-cell information transmission system involved in processes such as cell communication, cell migration, angiogenesis, and tumor cell growth (Figure 2) (Szwedowicz et al., 2022). In this review, we specifically address the use of EVs for the diagnosis and treatment of EC.

Specifically, the available data support the view that EVs could be leveraged as new targets for liquid biopsy in clinical practice can be understood through three aspects. First, as part of the tumor microenvironment, EVs mirror the biological essence of tumors. EVs released by cancer cells harbor tumor-specific markers that include tumor-specific proteins and mutated RNA sequences (Huang et al., 2019). Analysis of these biomarkers allows tumor evaluation without the need for direct tumor tissue acquisition, enabling early diagnosis. Second, EVs have huge potential in the transmission of information, permitting the study of DNA, RNA, and protein expression, which is more diverse than the research scope of single biomarkers, such as circulating tumor DNA. Third, as tumors progresses, the composition of EVs changes. For patients with mid-to late-stage EC, these alterations can be used to predict therapeutic biomarkers and aid clinical treatment.

Compared to other liquid biopsy biomarkers, EVs have many advantages as novel biomarkers. First, the concentration of EVs in the peripheral blood circulation of tumor patients is higher, with good accuracy and specificity (Wang et al., 2022). Second, the lipid bilayer structure of exosomes protects the contents carried within them from degradation. The abundance of microRNA in plasma exosomes remains stable even when stored at 4°C for 96 h or −80°C for an extended period (Kalluri and LeBleu, 2020). Third, patients harboring tumors accumulate proteins and nucleic acids in their body fluids through exosomes; most of the free DNA in their plasma is enriched in exosomes. These exosomes have been significantly correlated with the occurrence, development, metastasis, and drug resistance of tumors (Han et al., 2022). Fourth, EVs reflect the mutations and metabolic status of the source cells. For example, Exosomes loaded with CRISPR/Cas9 can target the mutant Kras^G12D^ oncogenic allele in pancreatic cancer cells, thereby suppressing proliferation and inhibiting tumor growth in syngeneic subcutaneous and orthotopic models of pancreatic cancer (McAndrews et al., 2021). Additionally, EVs mediated by cytosolic phospholipase A2 small interfering RNA and metformin can target and regulate the metabolism of glioblastoma. Thus, EVs are suitable for precise personalized treatment of tumors (Zhan et al., 2022).

## 3 Separation of exosomes and detection technology

The separation and detection of EVs are crucial aspects of related research. As the research deepens, new technological advancements continue to emerge. This section aims to discuss commonly used separation and detection techniques for EVs, and new techniques that have emerged in the past 3 years.

Ultracentrifugation is commonly used to recover EVs in high purity and yield (Martins et al., 2023). Ultrahigh-speed centrifugation methods include differential centrifugation and density gradient centrifugation (Roux et al., 2023). This method is suitable for the analysis of high-concentration biological samples. However, the pronounced heterogeneity of extracellular fluid may lead to non-ideal EV aggregation. Density gradient ultracentrifugation, as proposed by Zhao et al. (2022a), is considered the best method for separating many high-quality EVs. Size exclusion chromatography is based on EV size separation and is recommended in the latest (2023) release of the Minimal Information for Studies of Extracellular Vesicles (Welsh et al., 2024). This chromatography method is repeatable, cost-effective, and does not require specialized equipment, making it suitable for isolating EV (Sidhom et al., 2020). Ultrafiltration method utilizes ultrafine nanomembranes with different molecular weight cut off values to separate EVs from clinical samples or cell culture media. EVs and co-vesicles are based on large cell sizes. For example, Ko et al. (2023) introduced electrophoretic aspiration into tangent flow-driven ultrafiltration to solve the problems of clogging and contamination of ultrafiltration membrane pores. The approach separated exosomes with high concentration, purity, and intact morphology using electrophoretic aspiration assisted tangential flow. This report indicates that it may be possible to separate highly pure EVs using ultrafiltration.

Polymer-based precipitation methods have the advantages of simplicity, scalability, and high yield, making them suitable for the rapid diagnosis of diseases. Recently, many commercial reagent kits utilizing precipitation technology have been developed for the separation and enrichment of EVs. Examples of these kits include ExoQuick TC exosome precision solution, Total Exosome Isolation from cell culture media, and the ExoEasy Maxi Kit (Xiong et al., 2022). Gao et al. (2022) compared the ultrafiltration method with the ExoQuick TC precision method and found that size exclusion ultrafiltration resulted in higher yields, acceptable purity, and comparable biophysical properties and biological functions to the more expensive commercial precipitation method.

When traditional separation techniques cannot meet the requirements of exosome detection, the 2023 minimal information for studies guidelines of EVs suggest that combining detection techniques can enhance the detection of EVs. Combining immunoaffinity technology with magnetic separation efficiently enriches the recovery of EVs. For example, Pei et al. (2024) discovered that when titanium ions and antibodies were used in conjunction with magnetic nanoparticles, the captured EV protein content could reach 97 μg/mL. Additionally, 1,060 proteomes originating from exosomes were identified in the final isolated proteins. The MEIS microfluidic chip-based magnetically labeled exosome isolation system also greatly enhanced the enrichment of exosomes (Mun et al., 2023). The MEIS chip utilizes immunoaffinity antibodies to separate *her2* overexpressing exosomes from normal exosomes that lack disease-related information, making these isolated exosomes valuable for cancer diagnosis and monitoring (Mun et al., 2023). Microfluidic chips can be combined with immunoaffinity separation technology, as well as physical separation and enrichment methods. Liu et al. (2021) introduced the filter deterministic lateral displacement method to achieve high filtration flux and precise dimensional separation. By integrating a cascaded chip with a filter-deterministic lateral displacement structure, Liu et al. (2021) achieved outstanding separation efficiency (>96%), high cell purity (white blood cell removal rate of 99.995%), high cell viability (>98%), and a processing rate of 1 mL/min.

Finally, it is important to note that EV separation techniques are continuously evolving, and we may not be well versed in all their characteristics. Often, the method of separation selected may not meet the criteria for the target samples, leading to a lower purity of EVs (Yu et al., 2022). Therefore, we compiled and compared the operational mechanisms, advantages, and disadvantages of the EV separation technologies that have emerged in recent years (Table 1).

**TABLE 1:** Comparison of common extraction methods for extracellular vesicles.

Separation method	Mechanism	Advantage	Shortcoming
Differential centrifugation	Comprises several centrifugation steps aimed at removing cells, large vesicles, and debris, resulting in the precipitation of exosomes	A standard and widely used method for exosome separation	This method is less efficient when analyzing plasma and serum samples
Density gradient centrifugation	Involves the addition of separation media such as sucrose and iodixanol, allowing exosomes to remain at different gradient layers in solution	Enables the separation of low-density exosomes from other vesicles, particles, and contaminants	Highly sensitive to centrifugation time
Volume exclusion chromatography	Separates based on the size of the substances using columns filled with porous polymer beads	Allows precise separation of molecules based on size and application in different solutions	Time-consuming and not suitable for multiple samples
Filtration	Ultrafiltration membranes are used to separate exosomes from proteins and other macromolecules, concentrating the exosome population on the membrane	Filter allows the separation of small particles and soluble molecules from exosomes, enriching the exosomal population on the filter membrane	Exosomes may adhere to the filter membrane, leading to unnecessary loss. Additionally, exosomes may deform or be damaged
Polymer based precipitation	Involves mixing biological fluids with a polymer-containing precipitation solution, followed by incubation and low-speed centrifugation	Advantages of precipitation include mild conditions for exosome separation and use of neutral pH	Polymer-based precipitation methods may also separate non-vesicular contaminants, including lipoproteins
Immunoisolation	Utilizes various immunological methods, combining specific antibodies with magnetic beads for exosome separation	Allows for the separation of all exosomes or selective subtypes of exosomes. It is also suitable for qualitative and quantitative analysis of exosomal proteins	Not suitable for high-throughput sample processing
Microfluidic technology	Utilizes differences in size, density, and shape between exosomes and other cells and particles to achieve efficient separation and enrichment through microfluidic chips	Microfluidic chips enable automated separation of exosomes and offer high sensitivity for detecting low concentrations of exosomes	Higher costs, complex equipment, and high operational skill requirements
Magnetic separation method	Employs magnetic beads coated with magnetic materials that bind to specific molecules on the surface of exosomes, followed by separation using a magnetic field	Magnetic separation offers high reliability and reproducibility for exosome separation	Requires pre-treatment of samples to facilitate bead binding, which may introduce some error

After isolation, exosomes must be identified to analyze their roles in tumor growth, disease progression, and metastasis. Currently, exosome identification primarily depends on morphological characteristics, nanoparticle tracing analysis for particle size detection, protein blotting technology (Pan et al., 2023a), and flow cytometry to analyze exosome marker proteins such as CD9, CD63, CD81, and Heat Shock Protein 90 (HSP90) (Barranco et al., 2024; Huang et al., 2023). To provide a more comprehensive understanding of the current possibilities for identifying EVs, Table 2 compares and summarizes the commonly used methods. Additionally, we include examples that provide clear insights into the specific applications of EV identification analysis. Qi et al. (2023) used protein imprinting technology, nanoparticle tracking analysis and transmission electron microscopy to characterize exosomes. The authors suggested that exosomes containing Acyl-CoA synthetase long chain family member 4 play a role in the gemcitabine resistance of pancreatic cancer cells. Han et al. (2023) integrated flow cytometry analysis to demonstrate that tumor-derived exosomes carrying mir-1258-3p and miR-17-5p decreased T cell infiltration and activation, while increasing macrophage infiltration and M2 polarization, resulting in an immunosuppressive microenvironment.

**TABLE 2:** Comparison of common identification methods for exosomes.

Methods	Detection principle	Advantage	Shortcoming
Electron microscopy inspection	Scanning electron microscopy (SEM) or transmission electron microscopy (TEM)	SEM provides information on surface morphology; TEM allows observation of internal structure and morphology	Not suitable for high-throughput measurements; fixation processes can cause structural shrinkage of exosomes
Particle size and concentration detection	Dynamic light scattering and exosome particle tracing analysis (NTA)	Dynamic light scattering has a lower measurement limit of 10 nm and is highly sensitive to monodisperse particles. NTA allows direct, real-time observation of nanoparticles, accurately assessing both monodisperse and polydisperse samples	Dynamic light scattering is not suitable for measuring complex exosome samples with heterogeneous sizes. The measurement limit for NTA is only 70 nm
Western blotting	Surface marker detection	A mature method that can simultaneously detect multiple exosomal proteins with high sensitivity and specificity	Detected markers may vary depending on the cellular origin of the different types of exosomes
Flow cytometry	Detection of particle size and surface markers	Rapid and high-throughput, capable of analyzing particle size and volume with relatively low sample concentration requirements	Measurement limit is 400 nm (new digital flow cytometers can reach down to 100 nm)

Considering the heterogeneity and subtle differences in the performance of EVs, many exciting new separation and detection technologies are being explored. Xia et al. (2023) studied the DNA probe adsorbent manganese ion-modified black phosphorus (BP@Mn2+). The DNA hybrid nanosensor quickly distinguished exosomes derived from colorectal cancer cells from those derived from intestinal epithelial cells by sensing microRNAs. Jiang et al. (2024) developed a tetrahedral DNA (Apt TDNA) microelectrode sensor loaded with an adapter, which assisted the use of a polydopamine (PDA) coating with semiconductor properties to achieve single-particle-level sensitivity for cancer-derived exosome detection. Jang et al. (2021) developed recombinant exosomes loaded with photosensitizers.

## 4 Applications of exosomes for early diagnosis of EC

Tumor growth, invasion, and metastasis are regulated by bidirectional communication between cells and complex tissue environments. Tumor cells release EVs that shape the tumor microenvironment (Wang et al., 2023a), leading to changes and expansion that promote tumor development (Jin et al., 2022; Zhao et al., 2023). Because EVs can travel through body fluids such as blood and urine, they carry molecular information that reflects the biological status of various cell types, including tumor cells (Mishra et al., 2023; Zhou et al., 2023). Consequently, EVs have emerged as promising biomarkers with significant clinical relevance for early cancer detection, especially in cases where symptoms are not yet apparent (Chen et al., 2023b; Liang et al., 2023). For instance, Kim et al. (2023) demonstrated that using a combination of three miRNAs (miR-200b-3p, miR-3124-5p, and miR-92b-5p) significantly enhanced the diagnostic accuracy of small-cell lung cancer [area under the receiver operating characteristic curve (AUC, also abbreviated AUROC) = 0.93]. Additionally, Hinestrosa developed a classification system based on exocrine body markers that could detect stage I and II pancreatic, ovarian, and bladder cancers with high accuracy (95.5% for stage I pancreatic cancer, 74.4% for ovarian cancer, and 43.8% for bladder cancer, with a specificity >99% for all tumors screened) (Hinestrosa et al., 2022). Here, we focus primarily on the clinical application of EVs in the early diagnosis of EC.

Firstly, this study summarizes the specificity and sensitivity of various exosomal biomarkers for the early diagnosis of EC in recent years (Li et al., 2022a; Li et al., 2023b; Xing et al., 2017; Tong et al., 2015; Miyoshi et al., 2022) (Table 3). EVs can be utilized for the early diagnosis of EC owing to their involvement in malignant processes such as proliferation, angiogenesis, and metastasis. For instance, (Feng et al., 2023) discovered through protein mass spectrometry analysis that the interaction between p53 mutation G245S (p53-G245S) and heterogeneous nuclear ribonucleoprotein A2B1 (hnRNPA2B1) affects the formation of exosomes. Additionally, Liu et al. (Li et al., 2023b) revealed that p53 G245S mutation enhances ESCC proliferation and metastasis through hnRNPA2B1-ADP-ribosylation factor GTPase-activating protein 1 (AGAP1) mediated exosome formation. This study presented novel evidence of the molecular mechanism of EVs in ESCC progression, with AGAP1 mRNA potentially serving as a non-invasive biomarker for early ESCC detection. Furthermore, Zhang et al. (2022) identified that Poly(A) binding protein cytoplasmic 1 (PABPC1) is highly expressed in ESCC compared to normal esophageal epithelial tissue, suggesting its potential as a target for early EC diagnosis. Specifically, the expression of PABPC1 in ESCC positively correlates with tumor differentiation, and high PABPC1 expression in ESCC patients is associated with shorter median survival time post-surgery compared to low PABPC1 expression (*p* = 0.043) (Zhang et al., 2022). In addition, PABPC1 can enhance angiogenesis by mediating miR-21-5p expression and promoting the release and targeting of extracellular miR-21-5p to endothelial cells. Moreover, Rao et al. (2023) confirmed that the EV protein CD54 can be used as a biomarker for early EAC diagnosis. The available data indicate the promise of the use of tumor cell-secreted EVs as biomarkers for early EC detection as a novel clinical diagnostic approach.

**TABLE 3:** The application of different exosomal biomarkers in the early diagnosis of esophageal cancer.

Author (year)	Title	Exosomal biomarkers	Specificity	Sensitivity
Li et al., 2022	A signature of saliva-derived exosomal small RNAs as predicting biomarker for esophageal carcinoma: a multicenter prospective study	tsRNA sRESE	94.20%	90.50%
Zhang et al., 2023	Salivary Extracellular MicroRNAs for Early Detection and Prognostication of Esophageal Cancer: A Clinical Study	miR-1972 miR-6126 miR-1268a miR-4274 miR-4505 miR-4701-3p	91.04%	90.32%
Xing et al., 2017	Development and Validation of a Serum Biomarker Panel for the Detection of Esophageal Squamous Cell Carcinoma through RNA Transcriptome Sequencing	CHI3L1 MMP13 SPP1	72.14%	90%
Tong et al., 2015	Identification of the long non-coding RNA POU3F3 in plasma as a novel biomarker for diagnosis of esophageal squamous cell carcinoma	POU3F3 SCCA	81.4%	85.7%
Miyoshi et al., 2022	A microRNA-based liquid biopsy signature for the early detection of esophageal squamous cell carcinoma: a retrospective, prospective and multicenter study	miR-103 miR-106b miR-151 miR-17 miR-181a miR-21 miR-25 miR-93	89%	93%

EVs are found in nearly all bodily fluids including blood (Wang et al., 2023b), saliva (Xia et al., 2023b), and urine (Zhang et al., 2023), broadening the potential sources of liquid biopsy samples. In 2019, al.(Lin et al., 2019) revealed that salivary exosome GOLM1-NAA35 chimeric RNA (seG-NchiRNA) differentiates between tumor patients and healthy individuals, but also identify early stage tumor patients. A study published in 2022 reported that dual characteristic biomarkers (identified through small RNA Seq screening) from saliva-derived exosomes, tRNA GlyGCC-5 and sRESE, could distinguish patients with ESCC from the control group with high sensitivity (90.50%) and specificity (94.20%) (Li et al., 2022a). Additionally, the prognostic dual-feature risk score (RSP) proposed by Hao (Li et al., 2023b) could preoperatively predict that patients with high RSPs would benefit from postoperative adjuvant therapy. By continuing their research efforts, the team achieved significant breakthroughs. In July 2023, Hao et al. (Li et al., 2023b) published a clinical study on the early detection of salivary extracellular miRNAs for ESCC, with the goal of establishing miRNA profiles from salivary EVs and granules (EVPs) for early ESCC detection (Li et al., 2023b). In the study, we developed an early diagnostic model comprising six miRNAs. The model accurately identified all patients with ESCC in the training set (AUROC = 0.968) and successfully validated the results in two independent cohorts. Importantly, the model could differentiate patients with early stage (I/II) ESCC from the control group in the training (AUROC = 0.969, sensitivity = 92.00%, specificity = 89.17%), internal (sensitivity = 90.32%, specificity = 91.04%), and external (sensitivity = 91.07%, specificity = 88.06%) cohorts. Furthermore, the reproducibility of the six identified miRNAs ensured consistency in sample analysis and verification, which is critical for further validation in larger cohorts and potential integration into clinical practice.

Zhu et al. (2023b) initially conducted a proteomic analysis of plasma EVs and identified 419 proteins. Subsequently, they utilized bioinformatics techniques such as Gene Ontology and Kyoto Encyclopedia of Genes and Genomes enrichment analyses to identify differentially expressed proteins. The researchers have ultimately pinpointed a biomarker EV-CD14 that can be leveraged for the early detection of ESCC. In the test set comprising 30 cases, EV-CD14 demonstrated a high AUC value of 96% and an accuracy rate of 90% in predicting ESCC. Building on this research, Xing et al. (2017) investigated the RNA transcriptome of six pairs of ESCC tissues and identified three potential biomarkers: chitinase-3-like protein 1 (CHI3L1), matrix metallopeptidase 13 (MMP13), and osteopontin (SPP1). The AUC values for serum CHI3L1, MMP13, and SPP1 levels for differentiating patients with ESCC from controls were 0.732, 0.881, and 0.661, respectively. Combining these three proteins into a joint diagnostic model significantly enhanced the performance compared to individual markers, resulting in an AUC value of 0.928 (95% confidence interval CI 0.900–0.956), sensitivity of 90%, and specificity of 72.14% (Xing et al. 2017). The improved diagnostic efficacy of the combined model was subsequently validated. However, the use of a single exosome target for early EC detection presents limitations. Therefore, the association between solitary exosome targets and cancer assessment may not be sufficiently robust. Additionally, exosome protein mass spectrometry has inherent constraints and may not fully capture disease onset, making it susceptible to sample variations and methodological differences. Consequently, the primary objective at present is to identify multiple EV markers linked to cancer characterization and establish a comprehensive, multi-quantitative early diagnostic model. Tong et al. (2015) highlighted the diagnostic potential of plasma POU class 3 homeobox 3 (POU3F3) in ESCC (AUC 0.842; *p* < 0.001; sensitivity 72.8%; specificity 89.4%). When combined with Squamous Cell Carcinoma Antigen (SCCA), the diagnostic performance significantly improved, yielding an AUC of 0.926, sensitivity of 85.7%, and specificity of 81.4%. Notably, the combined detection of POU3F3 and SCCA effectively identified 80.8% of early ESCC cases (Tong et al., 2015).

Miyoshi et al. (2022) screened eight miRNAs (miR-103, miR-106b, miR-151, miR-17, miR-181a, miR-21, miR-25, and miR-93) that were overexpressed in ESCC serum samples from three miRNA expression datasets and constructed a circulating miRNA signature. Subsequently, the diagnostic performance of the ESCC model was validated in two independent cohorts [n = 126, AUC: 0.80 (95% CI: 0.69–0.91)]; and n = 165, AUC: 0.89 [95% CI: 0.83–0.94]. Importantly, the eight-miRNA model outperformed current clinical serological tumor markers (*p* < 0.001) in distinguishing early ESCC patients from healthy controls. Furthermore, Miyoshi et al. performed two prospective verifications confirming that the eight-miRNA combined diagnostic model can achieve good results in early screening of EC, with ideal diagnostic performance [n = 185, AUC: 0.92 (95% CI: 0.87–0.96)] and [n = 188, AUC: 0.93 (95% CI: 0.88–0.97)]. Larger scale studies with retrospective designs have been performed (Shin et al., 2023) and the prospective validation has been validated following retrospective studies. The data to date indicates the reliability and reproducibility of the foregoing research findings and will expedite the translation of research results into clinical applications.

Liquid biopsy has shown significant potential for EC. There have been numerous advancements in research on EVs in tumor diagnosis, with many scholars identifying EV markers for the diagnosis of EC. For example, the report that plasma EVs harboring miR-205-5p, miR-429, and miR-375-3p can be utilized as non-invasive biomarkers for early ESCC diagnosis (Kim et al., 2022) offers new perspectives and strategies for detecting esophageal tumors at an early stage. Looking ahead, the application of exosomes in clinical practice requires a shift towards prospective studies focused on high-risk EC populations. These studies should aim to differentiate patients who may develop EC in the future and guide precise personalized treatment strategies. For instance, Exosome Diagnostics, a leading industry in exosome diagnosis since 2008, released the midterm results of a prospective randomized study in May 2023 (Tutrone et al., 2023). This study reported that the ExoDx™ prostate test successfully stratified patients into low- and high-grade prostate cancer risk categories, assisting physicians in making informed decisions regarding the necessity of biopsies for patients with uncertain cancer risk based on prostate specific antigen results. Moreover, for men identified as having either extremely low or high prostate cancer risk as indicated by ExoDx, standard treatment protocols have proven vital in providing necessary information and care to ensure optimal patient outcomes (Tutrone et al., 2023).

## 5 Applications of exosomes in adjuvant therapy for EC

Treatment of EC has always posed a clinical challenge. Previous clinical data have revealed that patients with advanced EC have a 5-year survival rate of 30% (Wang et al., 2023c) with a median survival time of 25.9 months; tumor progression occurs in the majority (85.2%). Traditional treatment methods such as surgery, radiation therapy, and chemotherapy can partially control the condition. However, they have limitations and challenges (Triantafyllou and Wijnhoven, 2020), especially for advanced patients. Therefore, the discovery of new treatment strategies is imperative to enhance the survival rate of patients with EC. In recent years, EVs have garnered attention as potential therapeutic agents. These vesicles regulate inflammatory responses, angiogenesis, and fibroblast activation (Xie et al., 2021; Jin et al., 2021). They also create a conducive growth environment for tumor cells (Li et al., 2021b) and aid in evading immune system attacks (Zhao et al., 2022b), thereby fostering tumor cell proliferation, invasion, metastasis, and drug resistance (Lin et al., 2022; Akoto and Saini, 2021). This section delves into the clinical applications and potential mechanisms of EVs in the treatment of EC.

EVs are primarily responsible for transmitting information between tumor cells and can be likened to “couriers.” As new targets for cancer-targeted therapy, tumor exosomes can be suppressed in several ways: One method is to directly prevent the couriers from leaving, which involves disrupting the synthesis and secretion of EVs. For example, Zhao et al. (2021b) discovered that interleukin-6 (IL-6) secreted by cancer-associated fibroblasts (CAF) and miRNA-21 (miR-21) packaged in exosomes activate IL-6 signaling and transcription activating factor 3 (STAT3), leading to cisplatin resistance in patients with ESCC. Therefore, inhibiting the paracrine and autocrine secretion of CAF-related IL-6 is a potential target for reversing drug resistance. Secondly, if the exosomes cannot be intercepted during their formation, obstacles can be placed on their delivery route. For instance, Jin et al. (2021) found that blocking the phosphoinositide 3-kinase/AKT and β-catenin signaling pathways derived from exosomal miR-3656 can inhibit the growth and metastasis of ESCC cells. Additionally, Qiao et al. (2023) described the significant reduction of the tumor-promoting effect of LINC01592 tumor-associated macrophages by disrupting the E2F6/NBR1/MHC-I signaling axis derived from exosomes of LINC01592. Interestingly, the authors also discovered that inhibiting LINC01592 can also lead to increased major histocompatibility complex class I (MHC-I) expression on the surface of tumor cells, thereby enhancing the effectiveness of CD8^+^ T cell reinfusion.

Owing to the protection of the phospholipid bilayer, EVs have good stability (Arya et al., 2024; Liu et al., 2024a) and can protect their internal payload. Therefore, EV testing can be used to guide the prognosis of patients with EC. Zhu et al. (2021) analyzed the metabolomic patterns of EVs in recurrent and non-recurrent patients after esophagectomy using machine learning and identified a panel of extracellular vesicle metabolic biomarkers (3′-UMP, palmitic acid, palmitol, and isobutyl decanoate) for predicting the recurrence of ESCC with 98% accuracy. These metabolomic features exhibit significant differential changes throughout all ESCC stages and are likely associated with cancer metabolism, potentially serving as novel biomarkers for ESCC prognosis. Li et al. (2022) suggested that miR-200a promotes the proliferation, migration, and invasion of EC cells by altering the Keap1/Nrf2 signaling pathway. The authors reported that miR-200a transported via EVs can be a valuable biomarker for predicting the prognosis of patients with ESCC. Additionally, Luo et al. (2016) found that long noncoding RNAs (lncRNAs) AFAP1-AS1 promotes the *in vitro* proliferation of esophageal cancer cells, with expression observed in both the nucleus and cytoplasm, indicating that AFAP1-AS1 is predominantly localized within the nucleus, thereby enhancing its potential role in epigenetic or transcriptional regulation. The authors suggest that AFAP1-AS1 may influence the proliferation of ESCC cells through epigenetic regulation of Homeobox B7 (HOXB7) and by interacting with Polycomb Repressive Complex 2 (PRC2). Consequently, Luo et al. (2016) concluded that the upregulation of exosomal lncRNA AFAP1-AS1 in ESCC is closely associated with shorter progression-free survival and unfavorable overall survival.

The exosome miRNA model established by Liu et al. (2020) can effectively identify patients with lymph node metastasis in ESCC and is significantly superior to preoperative X-ray computed tomography. This miRNA model has the potential to predict preoperative lymph node metastasis in ESCC patients, assist thoracic surgeons in determining appropriate treatment plans, and enhance patient prognosis. Zhang et al. (2024) quantified the plasma exosome concentrations of patients with locally advanced ESCC at two time points: before radiotherapy and chemotherapy, and 2 months after treatment. A significant decrease in average exosome concentration from 7.3 × 10^11^ particles/mL before treatment to 5.4 × 10^11^ particles/mL after treatment was evident (*p* < 0.001). Using multivariate Cox regression analysis, the authors further demonstrated that dynamic changes in exosome levels could serve as independent predictors of progression-free survival. Liu et al. (2020b) proposed that serum exosome miR-766-3p could act as a prognostic indicator for evaluating ESCC. The findings validated the correlation between Tumor, Node, Metastasis (TNM) staging and elevated levels of exosomal miR-766-3p in ESCC patients (*p* < 0.05), with higher levels of miR-766-3p expression in serum exosomes indicating a poorer prognosis, with hazard ratios [95% CI] for overall survival of 2.21 [1.00, 4.87] and disease-free survival of 2.15 [1.01, 4.59].

Exosomes have a dual effect on tumors. In addition to inhibiting the immune system and promoting tumor development, tumor exosomes can improve the efficacy of adjuvant therapy. Chen et al. (2020) found that overexpression of miR-450a-5p can increase radiation sensitivity in patients with ESCC and could be a potential new therapeutic target to reduce radiation therapy resistance. Similarly, Du et al. (2022) found that inhibiting the high expression of plasma exosomes harboring High mobility group box 1 protein can reduce radiation-related adverse reactions in patients with ESCC. Sun et al. (2021) found that miR-199a-5p enhances the radiosensitivity of radiation-resistant cells by targeting Endonuclease/exonuclease/phosphatase family domain-containing protein 1 and inhibiting the Ataxia-telangiectasia and Rad3-related protein/Checkpoint kinase 1 signaling pathway. However, lncRNA-NORAD inhibits miR-199a-5p expression by competitively binding to PUM1 from pri-miR-199a1, thereby rendering ESCC patients resistant to radiotherapy. Finally, they confirmed that lncRNA activated by deoxyribonucleic acid damage (lncRNA-NORAD) is a potential therapeutic target for improving the efficacy of ESCC immunotherapy combined with radiation therapy. Luo et al. (2019) found that high concentrations of miR-339-5p in serum are positively correlated with high sensitivity to radiotherapy and favorable survival rates. Additionally, they observed that miR-339-5p can predict the pathological response to preoperative radiotherapy in locally advanced ESCC, as miR-339-5p expression was significantly downregulated in the T3/T4 stage compared to the T1/T2 stage in ESCC patients (*p* < 0.05). Mechanistically, miR-339-5p enhances radiosensitivity by targeting Cdc25A and is transcriptionally regulated by Runx3. Therefore, miR-339-5p may serve as a promising non-invasive biomarker for facilitating personalized treatment.

**FIGURE 3:**
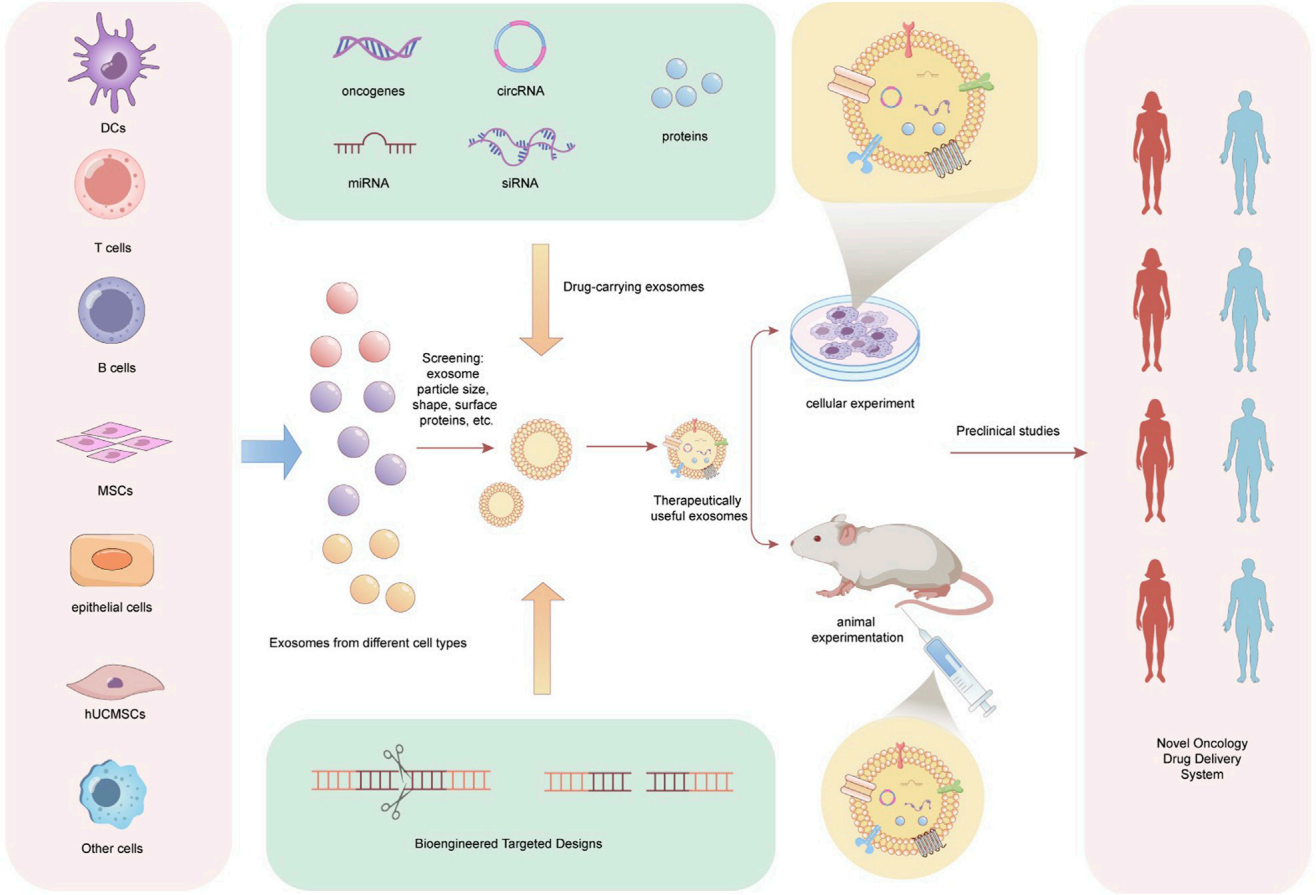
The clinical application prospect of exosomes as a new generation of drug delivery vehicles. Exosomes are collected from various cell types, including dendritic cells (DCs), T cells, B cells, mesenchymal stem cells (MSCs), and epithelial cells. These exosomes are screened based on characteristics such as size, dimensions, and surface proteins to identify suitable candidates. Utilizing bioengineering targeting technologies, different drug-loaded exosomes are designed, incorporating agents such as oncogenes, circRNA, miRNA, siRNA, and proteins. Upon obtaining exosomes with therapeutic potential, *in vitro* experiments (cell-based assays) and *in vivo* studies (mouse models) are conducted to evaluate the safety and efficacy of the drug-loaded exosomes. Furthermore, prior to the comprehensive clinical application of these drug-loaded exosomes, preclinical studies must be performed in relevant populations. Ultimately, the resulting drug-loaded exosomes are poised to serve as a novel tumor drug delivery system. DCs, Dendritic Cells, T cells, Thymus Dependent Lymphocytes, B cells, Bursa Dependent Lymphocytes, MSCs, Mesenchymal Stem Cells, hUCMSCs, human Umbilical cord Mesenchymal Stem Cells.

Recently, EV drug delivery has become a major research focus. The main advantages of using EVs as drug delivery carriers are their ability to lower drug concentration, accurately target the site of injury, enhance efficiency, and reduce drug toxicity and side effects (Tian et al., 2023; Chen et al., 2023c). Accordingly, this review also discusses the potential applications of EV drug delivery in EC (Figure 3). Ma et al. (2022) engineered exosomes produced by M1 macrophages (M1Exos) to address the triple challenges of the tumor immune suppression microenvironment, hypoxic microenvironment, and rapid DNA damage repair occurring following radiotherapy. Additionally, the authors described that the outer membrane of M1Exos contains nanoantibodies targeting programmed death-ligand 1, which can alleviate CD8^+^ T cell immunosuppression and reshape the immunosuppressive microenvironment of tumors. Moreover, exosomes carrying miR-375 from human umbilical cord mesenchymal stem cells can inhibit the expression of ENAH actin regulator 1, thus hindering the progression of ESCC (He et al., 2020). To identify differentially expressed miRNAs in cancerous versus adjacent tissues from various public miRNA databases, the authors additionally aimed to validate their functionality through experiments involving exosomes and critical biological pathways. The encapsulation of artificially designed miR-375 molecules in exosomes could open new avenues for ESCC drug development. Similarly, as suggested by He et al. (2020) and Chen et al. (2023d), miR-100-5p inhibits the proliferation, migration, invasion, and tube formation of tumor-associated lymphatic endothelial cells (TLECs). Furthermore, miR-100-5p also suppresses lymphangiogenesis in ESCC xenografts. Mechanistically, this inhibitory effect is mediated by the miR-100-5p-induced suppression of the IGF1R/PI3K/AKT signaling axis. Therefore, taken together, CAF-derived exosomal miR-100-5p can target the IGF1R/PI3K/AKT axis to inhibit lymphatic metastasis in ESCC. Consequently, exosomal miR-100-5p also holds potential as a drug delivery vehicle in the development of therapies aimed at suppressing ESCC progression. Furthermore, this study provides an overview of current extracellular vesicle drug delivery methods, encompassing small molecule drug delivery, including ultrasound (Wang et al., 2019), mixed drug delivery (Kim et al., 2016), extracellular vesicle peptide modifications (Shen et al., 2022), EV delivery of nucleic acids that include small interfering RNA, miRNA, and plasmids (Geng et al., 2023), and EV delivery of macromolecule such as chitosan oligosaccharide (Li et al., 2021c).

## 6 Future prospects

As one of the three categories of liquid biopsy, EVs are abundant in body fluids and carry various biological information molecules from secreted cells. The double phospholipid layer membrane structure provides better protection for the contents, making them potential biomarkers. An advantage of exosomes is their abundance (Li et al., 2023c; Ten et al., 2024). Tumor cells can continuously secrete exosomes and enter the circulatory system during their growth process, and their high metabolic rate leads to a much higher production of exosomes than that of normal cells (Vahabi et al., 2023; Liu et al., 2024b). Additionally, compared to the dependence of circulating tumor DNA generation on tumor cell death, exosomes secreted by living cells represent a continuous event that can reflect the real-time progression of tumor development (Claridge et al., 2021; Liu et al., 2022). However, it is important to note that current research on the mechanism of action of EVs has mainly involved *in vitro* experimental systems and animal models (Xu et al., 2023; Pan et al., 2023b). There is no consensus on experimental methods for extracting and quantifying EVs from cell culture supernatants. As a result, research on EV-related functions only simulates an almost *in vivo* environment, and individual differences can lead to heterogeneity in the functions of extracellular vesicles. Therefore, further in-depth studies are required to elucidate the mechanisms of EV formation and secretion.

Furthermore, although exosomes have demonstrated clinical value in the diagnosis and treatment of esophageal cancer, their safety issues warrant attention. Exosomes may elicit potential immune responses, as their components could be recognized by the host immune system as foreign substances, activating immune reactions that may lead to adverse effects (Xia et al., 2024). Additionally, the non-target effects of exosomes should not be overlooked; their widespread distribution could impact various cell types and tissues, inadvertently delivering therapeutics to non-target cells and thereby causing side effects (Fusco et al., 2024). Current data on long-term safety remain limited, and the metabolic and clearance mechanisms of exosomes within the body are not yet fully understood, posing potential risks for their application in the long-term treatment of esophageal cancer. Therefore, establishing rigorous standardized protocols and regulatory frameworks to ensure the safety and efficacy of exosome extraction, characterization, and application is of significant importance.

To facilitate the transition of exosomes from basic research to clinical application, this study suggests focusing on the following three aspects. First, given the heterogeneity of exosomes and their close relationship with tumor heterogeneity, subtypes, and therapeutic detection, it is essential to develop more single-exosome detection technologies. Second, future research should stratify patients (for example, based on cancer risk stratification, pathological staging, etc.) using established standardized exosome research methods to ensure the reproducibility and comparability of experiments, which will aid in advancing the application of exosome-related molecules in esophageal cancer. Third, to enable the large-scale clinical application of exosomes in the future, subsequent studies could place greater emphasis on the diagnostic value of multi-molecule combined detection models involving exosomes in esophageal cancer, while conducting more extensive clinical trials to validate the actual efficacy of exosomes in this context.

## 7 Conclusion

In recent years, exosomes have been extensively studied and have demonstrated clinical value in various cancers. For instance, in prostate cancer, exosomes have been found to carry specific proteins and small RNAs that are closely associated with the onset and progression of the disease (Canovai et al., 2023; Atri et al., 2024). Similarly, in esophageal cancer, some studies have identified small RNAs such as LncRNA RP11-465B22.8 within exosomes as potential biomarkers (Hu et al., 2021). In lung cancer, exosomes promote tumor cell evasion and metastasis by delivering immune regulatory factors (Wang et al., 2024). Preliminary data also suggest that esophageal cancer cells may utilize exosomes to alter the surrounding microenvironment, thereby facilitating aggressive behavior (Ning et al., 2023). However, further exploration of the role of exosomes in the microenvironment of esophageal cancer is necessary to reveal new therapeutic targets.

In the competitive landscape of exosome diagnosis, there have been some breakthrough achievements and technological developments in industrial research on exosome diagnosis internationally. Exosome Diagnostics has approved multiple products for market launch, such as the ExoDxLung (ALK) lung cancer diagnostic kit and the ExoDx Prostate (IntelliScore) blood/urine detection kit. These products can be used to analyze RNA, proteins, and other components in exosomes obtained from the blood or urine samples of patients to accurately diagnose cancer. Furthermore, tumor-related exosomes can exert various biological functions through biological barriers, making them a promising new route for drug delivery and gene therapy vectors (Peswani Sajnani et al., 2021). Owing to their highly complex and variable composition, the cellular specificity and biological effects of EVs are unpredictable. When used as drug carriers, it is essential to improve cell specificity and circulation time, increase accumulation in targeted tissues, and enhance carrier delivery. This is a new trend for EVs as targeted drugs for disease treatment, providing compelling advantages and prospects for treating radiation damage.
